# Two-tailed RT-qPCR panel for quality control of circulating microRNA studies

**DOI:** 10.1038/s41598-019-40513-w

**Published:** 2019-03-12

**Authors:** Peter Androvic, Nataliya Romanyuk, Lucia Urdzikova-Machova, Eva Rohlova, Mikael Kubista, Lukas Valihrach

**Affiliations:** 1 Institute of Biotechnology of the Czech Academy of Sciences - BIOCEV, Vestec, 252 50 Czech Republic; 2 Laboratory of Growth Regulators, Faculty of Science, Palacky University, Olomouc, 78371 Czech Republic; 3 Institute of Experimental Medicine of the Czech Academy of Sciences, Prague, 142 20 Czech Republic; 4 TATAA Biocenter AB, Gothenburg, 411 03 Sweden; 5 Department of Anthropology and Human Genetics, Faculty of Science, Charles University, Prague, 128 43 Czech Republic

## Abstract

Circulating cell-free microRNAs are promising candidates for minimally invasive clinical biomarkers for the diagnosis, prognosis and monitoring of many human diseases. Despite substantial efforts invested in the field, the research so far has failed to deliver expected results. One of the contributing factors is general lack of agreement between various studies, partly due to the considerable technical challenges accompanying the workflow. Pre-analytical variables including sample collection, RNA isolation, and quantification are sources of bias that may hamper biological interpretation of the results. Here, we present a Two-tailed RT-qPCR panel for quality control, monitoring of technical performance, and optimization of microRNA profiling experiments from biofluid samples. The Two-tailed QC (quality control) panel is based on two sets of synthetic spike-in molecules and three endogenous microRNAs that are quantified with the highly specific Two-tailed RT-qPCR technology. The QC panel is a cost-effective way to assess quality of isolated microRNA, degree of inhibition, and erythrocyte contamination to ensure technical soundness of the obtained results. We provide assay sequences, detailed experimental protocol and guide to data interpretation. The application of the QC panel is demonstrated on the optimization of RNA isolation from biofluids with the miRNeasy Serum/Plasma Advanced Kit (Qiagen).

## Introduction

Circulating cell-free microRNAs have emerged in recent years as promising candidates for minimally invasive clinical biomarkers for diagnosis, prognosis, and monitoring of a multitude of human pathologies^1–6^. After this recognition, a massive wave of research aiming at identifying disease-associated microRNAs followed. A search for the keywords “microRNA”, “biomarker” and “blood” returns over 5000 hits in the PubMed database (September 2018) with the number of studies increasing every year. Despite promising advances in the field (www.clinicaltrials.gov), there is still no microRNA test in clinical practice^7,8^. There are many reasons behind the current unsatisfactory state and their comprehensive discussion is beyond the scope of this article (for reviews see^8,9^). One issue is the poor agreement between studies, which may in part be attributed to the lack of standardization^10^ and technical difficulties associated with the workflow^11,12^. Protocols for blood collection, sample processing, storage, RNA isolation, and microRNA quantification often vary across laboratories leading to discordant results^11,13,14^. Efforts are ongoing to standardize the blood sampling and processing steps to improve the reproducibility of microRNA analyses^15–18^ (www.cancer-id.eu; www.spidia.eu). However, notable sources of variation remain. These include the RNA isolation, co-purification of inhibitors of enzymatic reactions, and cellular contamination of the biofluid samples^19–21^. These factors may bias the measured microRNA profiles leading to false-positive discoveries of disease-associated biomarkers. Rigorous control of sample quality and technical workflow is therefore of highest importance.

An efficient way to monitor technical variation is the addition of exogenous spike-in molecules prior to RNA isolation^22–24^. The signal from the spike-ins reflect yields and extraction efficiency, which identifies abnormal samples that should be reanalysed or disqualified. A second set of exogenous spike-ins can be added before the microRNA quantification to control for bias introduced downstream of this step, such as the inhibition of enzymatic reactions. This concept has been described previously^20,22,23^ and is also available as a commercial product (e.g. RNA Spike-In Kit, for RT marketed by Qiagen). Yet, to our knowledge, there is no tool to perform such extended quality control on challenging experimental samples described in detail in literature.

Here, we present the Two-tailed quality control (QC) panel; a tool to assess the technical quality of RNA isolation, degree of inhibition and erythrocyte contamination, primarily in liquid biopsy samples, such as serum and plasma. The panel is based on Two-tailed RT-qPCR; a highly specific method for microRNA quantification^25^. We provide detailed experimental protocol, guide to data interpretation, and sequences of the RNA oligonucleotides and RT-qPCR assays used (Supplementary file) that can be ordered from any licensed oligo manufacturer. The QC panel is based on standard reagents and is intended to provide researchers a convenient tool to assess technical performance and quality of the samples before investing resources into extensive quantification experiment, such as small-RNA sequencing or high-throughput RT-qPCR. We demonstrate its utility by optimizing an RNA isolation protocol, screening for haemolysis, and testing for outliers with compromised quality. We also report data obtained with the recently launched miRNeasy Serum/Plasma Advanced Kit (Qiagen) for two biofluids collected from human and rat.

## Results

### Design of the QC panel

**Figure 1:**
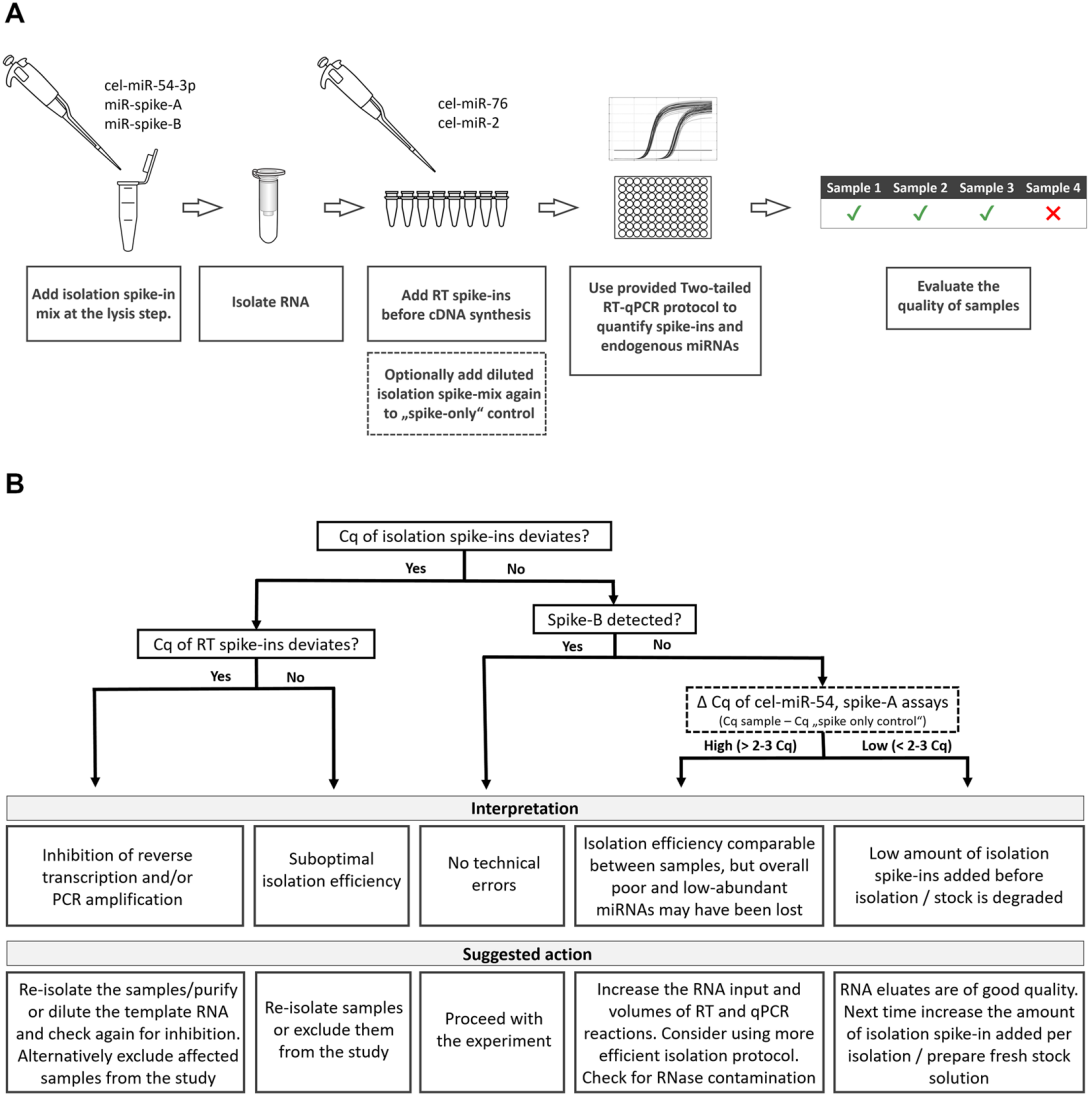
QC workflow with the Two-tailed QC panel. **(A)** A mix of three synthetic RNA spike-ins (cel-miR-54, spike-A, spike-B) is added prior to RNA isolation from the biofluid sample. A second mix of two spike-ins (cel-miR-76, cel-miR-2) is added before cDNA synthesis step. Optionally, a diluted isolation spike-in mix is used as a template in a “spike-only” control reaction to determine spike-in baseline signal (for details see Supplementary file section 3.2.2). Two-tailed RT-qPCR is used to quantify the spike-ins along with three endogenous microRNAs (let-7a, miR-23a and miR-451a) to evaluate the technical quality of RNA isolation, effect of inhibition and the level of haemolysis. (**B)** Decision chart for data interpretation and troubleshooting (see also Supplementary file section 4).

The Two-tailed QC panel is composed of five synthetic spike-in microRNAs and eight Two-tailed assays targeting these synthetic spike-ins, and three endogenous microRNAs (Fig. 1). The spike-ins are based on *C*. *elegans* microRNAs and artificial sequences and have no significant homology to any known human, mouse or rat microRNA (Table 1). All spike-ins have 5′ terminal phosphate to mimic endogenous microRNAs, and to allow incorporation into microRNA libraries for Next Generation Sequencing (NGS).

**Table 1:** Synthetic RNA spike-ins used in the Two-tailed QC panel.

Usage	Name	Sequence	GC %	Origin
Isolation spike-ins	cel-miR-54-3p	/5Phos/UACCCGUAAUCUUCAUAAUCCGAG	41.7	*C*. *elegans*
	miR-spike-A	/5Phos/UGCAGCCCUACCGACACGUUCC	63.6	artificial
	miR-spike-B	/5Phos/ACUCAGGUUGUAGGAGCGGUCUU	52.2	artificial
RT spike-ins	cel-miR-76-3p	/5Phos/UUCGUUGUUGAUGAAGCCUUGA	40.9	*C*. *elegans*
	cel-miR-2-3p	/5Phos/UAUCACAGCCAGCUUUGAUGUGC	47.8	*C*. *elegans*

Three spike-in RNAs (cel-miR-54, spike-A and spike-B) comprise the isolation spike-in mix and are added to the samples at a known constant amount prior to RNA isolation, serving as control for the technical performance of the RNA isolation protocol (Fig. 1A). The three spike-ins have varying GC content (41.7–63.6%) and are present at concentrations reflecting high (cel-miR-54, 1e + 7 copies/μl), moderate (spike-A, 2e + 5 copies/μl), and low (spike-B, 4e + 3 copies/μl) abundant microRNAs (Supplementary file). The ΔCq’s between the isolation spike-ins should, in absence of inhibition, be in the range 3.5–5.5 cycles (accounting for differences in RT-PCR efficiencies of the Two-tailed assays), however, these values may be influenced differently by individual isolation protocols due to various biases^26,27^.

Two RNA spike-ins (cel-miR-76 and cel-miR-2) comprise the reverse transcription (RT) spike-in mix and are added to the RT reaction serving as controls for cDNA synthesis, PCR amplification and as general controls for the presence of inhibitors in RNA eluates (Fig. 1A). Cel-miR-76 (1e + 7 copies/μl) is added at 100x higher concentration than cel-miR-2 (1e + 5 copies/μl) and their ΔCq should be 5.5–6.5 cycles (accounting for differences in PCR efficiency of the Two-tailed assays).

The QC panel also contains assays for the three endogenous microRNAs: let-7a, miR-23a and miR-451a. Let-7a is abundant in plasma and serum^20,28,29^ and serves as positive control. Mir-23a is also abundant in plasma/serum and its level is independent of haemolysis, while miR-451a is highly abundant in erythrocytes and its level increases dramatically upon haemolysis^20,30^. The ΔCq (mir-23a – mir-451a) indicates degree of haemolysis in the samples^20^.

### Optimization of sample input volume

**Figure 2:**
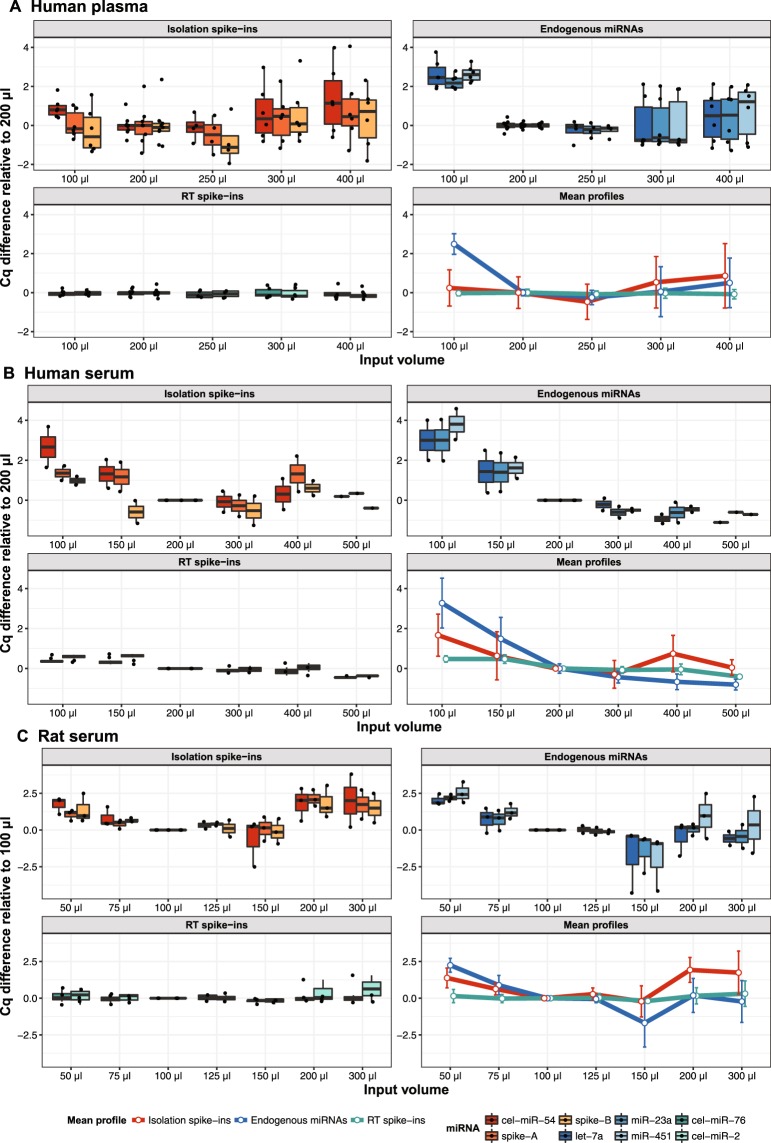
Optimizing input volumes of (**A**) human plasma, (**B**) human serum, and (**C**) rat serum for RNA isolation. Data are presented as ΔCq between Cqs obtained with the tested volume and an input volume of 200 μl (human) or 100 μl (rat). Each dot is one isolation replicate. Optimum starting serum/plasma volumes based on absolute endogenous microRNA yields are 250 μl for human plasma, 300–500 μl for human serum, and 150 μl for rat serum (blue mean profiles). Error bars on mean profiles panels indicate standard deviation (SD).

A factor that is often neglected, but can have major impact on the quality of microRNA quantification data, is the initial input volume used for the RNA isolation^13,26^. Liquid biopsy samples contain very low amounts of microRNAs and researchers may be tempted to use as much sample material as possible for RNA isolation. However, with increasing amount of starting material risk of carryover of contaminating substances and saturation of the purification column increases^31,32^. Most commercial RNA isolation kit manufacturers recommend 200 μl starting serum/plasma volume, however, optimum volume depends on the isolation protocol, sample type and also organism^26^. Optimizing the sample volume is therefore recommended when setting up a new isolation protocol or extracting a new type of sample. For such optimization the Two-tailed QC panel is a tool to assess relative isolation efficiency, absolute yield, and test for the presence of inhibitors to decide the optimal input volume. With this strategy we optimized protocol based on the miRNeasy Serum/Plasma Advanced Kit (Qiagen) for RT-qPCR analysis of human plasma, human serum, and rat serum (Fig. 2).

We found a non-linear relation between the input sample volume and cDNA yield as reflected by RT-qPCR signal of endogenous microRNAs (Fig. 2). The non-linearity is caused neither by RT nor PCR inhibition, as the signals from the RT spike-ins were independent of volume. Rather the non-linear response is due to variations in RNA isolation efficiency, as reflected by the RT-qPCR response of the isolation spike-ins (Fig. 2). We observed poor isolation efficiency with low input volumes (<200 μl for human, <100 μl for rat), but also with higher input volumes (≥300 μl for human, ≥200 μl for rat), where the response was also more variable (Fig. 2). Based on our results, optimum starting sample volumes with our workflow are: 250 μl for human plasma, 300–500 μl for human serum, and 150 μl for rat serum.

### Assessing the effect of co-precipitants in the isolation procedure

**Figure 3:**
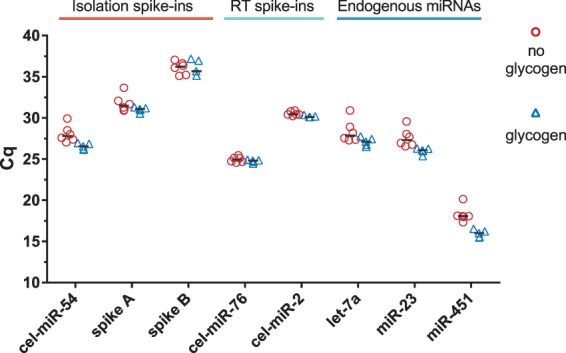
Effect of glycogen carrier on microRNA quantification in human plasma. Identical sample aliquots were isolated with (n = 5) or without (n = 6) addition of glycogen carrier starting from 200 μl, and quantified with the Two-tailed QC panel. Extractions with glycogen had significantly higher yields (average difference between Cq means: 1.25 cycles; paired T-test p = 0.011) and higher reproducibility (F-test, p < 0.001).

Since biofluids like serum and plasma contain very low amounts of RNA, significant portion may be lost during the isolation procedure due to adsorption to the pipette tips, tube walls etc. Losses can be reduced by adding carriers such as MS2 phage RNA or yeast tRNA to the samples before RNA isolation^33,34^. However, RNA-based carriers are less suited when NGS is used for downstream analysis as the exogenous RNAs may consume sequencing reads. Other carriers, such as linear acrylamide, BSA or glycogen may then be used instead^24^. Using the Two-tailed QC panel we tested the impact of using glycogen as carrier in our isolation procedure (Fig. 3). In accordance with previous observations^33,34^, we found that addition of glycogen significantly improved the reproducibility of isolation (F-test, p < 0.001) and significantly increased the yield (average Cq difference 1.25; paired T-test p = 0.011) with no negative effects on the downstream RT-qPCR analysis (Fig. 3). Based on these findings, we recommend addition of glycogen to increase the robustness and efficiency of microRNA isolation with the miRNeasy Serum/Plasma Advanced Kit (Qiagen).

### Assessing the level of haemolysis in serum/plasma samples

A major complication in microRNA analysis of serum/plasma samples is contamination with microRNAs derived from lysed blood cells^20,30,35^ and in particular haemolysed erythrocytes. Plasma and serum samples should therefore be assessed for haemolysis. Standard method is to measure absorption at 414 nm, 540 nm and 578 nm, which are the absorption peaks of free oxyhemoglobin^36^. An alternative approach, which is applicable also when the original sample is no longer available, is to measure the ratio of miR-23a, which is insensitive to haemolysis, and miR-451a, which is highly enriched in erythrocytes^20^. Blondal *et al*.^20^ established threshold ΔCq (miR-23a–miR-451a) values as quality indicators for human samples: ΔCq > 5 indicates there may be erythrocyte contamination, and ΔCq > 7 indicates high risk of haemolysis. A complication, however, is that the indicator is sensitive to the relative isolation yields of miR-23a and miR-451, as well as their relative RT yields and PCR efficiencies of the assays used to quantify them. Hence, the threshold ΔCq’s reported by Blondal *et al*.^20^ are valid only for their particular workflow and protocol, and should not be used as general indicators. Here, we establish threshold ΔCq values for the Two-tailed QC panel and our recommended workflows (Supplementary file).

**Figure 4:**
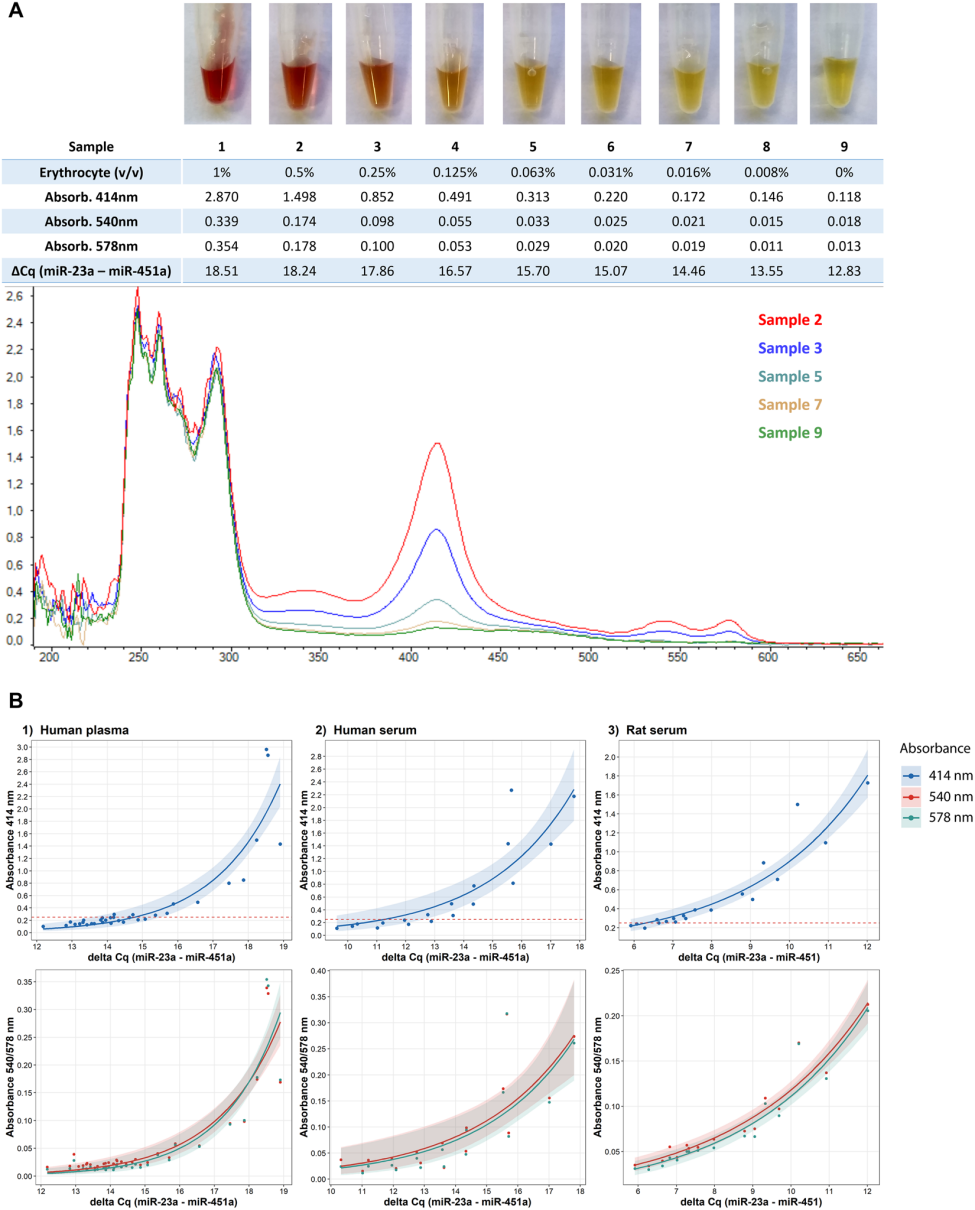
Assessing haemolysis in serum/plasma samples. **(A)** Human plasma samples with varying degree of haemolysis, corresponding A_414_, ΔCq (miR-23a–miR-451a), and selected UV-Vis spectra. (**B)** Correlation between ΔCq (miR-23a–miR-451a) and A_414_, A_540_ and A_578_, respectively. Exponential regression line with 95% confidence interval is shown. Dashed red line indicates A_414_ = 0.25 as threshold for non-haemolysed samples. Corresponding ΔCq thresholds are ~15 (human plasma), ~11 (human serum), and ~6 (rat serum).

We prepared duplicate haemolysis dilution series for each sample type and constructed standard curves to correlate ΔCq (miR-23a – miR-451a) values to absorbance at 414 nm (Fig. 4B). To increase the number of data points, samples screened in other experiments with the same workflow were also included. Correlation between A_414_ and linear transformation of ΔCq (2^ΔCq^) is significant for all three biofluids (Pearson r ≥ 0.80, p < 0.0001). A_414_ for plasma sample 1 was outside the linear range of the absorption spectrophotometer and was estimated by interpolation. A_540_ and A_578_ nm dependences show the same trend, although those peaks are considerably less significant in the absorbance spectrum (Fig. 4A). A_414_ ≤ 0.2–0.25 has previously been recommended as threshold for non-haemolysed samples^13,20,35^. Based on our calibration this corresponds to a ΔCq of 15 cycles for human plasma, 11 cycles for human serum, and 6 cycles for rat serum for our workflow (Fig. 4B; Supplementary file).

## Discussion

We present an RT-qPCR based protocol to assess the technical performance of workflows for analysis of microRNAs in body fluids such as serum and plasma. The QC panel developed is based on two sets of synthetic spike-in molecules and three endogenous microRNAs to assess RNA isolation yield, RT yield, PCR efficiency, and haemolysis (Fig. 1).

A highly error-prone step in microRNA analysis workflow is RNA isolation. Several studies have studied the effect of input volume on microRNA recovery, reporting varying results^26,37,38^. However, consistent observations are substantial variability between replicate isolations and non-linear dependence of the input volume on the amount of microRNAs detected^26,31^. Here, we studied the effect of input sample volume when extracting with the recently launched miRNeasy Serum/Plasma Advanced Kit from Qiagen and found that optimal input volume is different for the three sample types: human plasma, human serum, and rat serum (Fig. 2). We also found that higher input volumes (>300 μl for human, >150 μl for rat), although still in the range recommended by the manufacturer, lead to less reproducible Cq values compared to moderate input volumes (200–300 μl for human, 100–150 μl for rat). Using spike-in controls we showed this is due to inhibition of neither cDNA synthesis nor PCR, as suggested previously^20^, but rather to impaired isolation efficiency, possibly because of saturation of the purification column. We confirm previous observations that adding a carrier improves extraction yield and reproducibility^32–34,39^. We also show glycogen is a suitable alternative to RNA-based carriers when using the miRNeasy Serum/Plasma Advanced Kit (Qiagen) conferring advantage when samples shall be analysed with NGS (Fig. 3).

Another contribution to bias is microRNAs from leaking blood cells^30,35^. While cellular contamination can be minimized by careful removal of the plasma fraction and dual centrifugation to efficiently remove platelets, haemolysis remains a problem. Haemolysis can occur during sampling and handling procedures and the released cellular microRNAs distort the measured microRNA profiles, which no longer reflect exclusively cell free microRNA^30,35,36^. This not only hampers biological interpretation of the results, but can distort normalization or RT-qPCR data. For example, miR-16-5p is widely used as reference microRNA^9^, but it is also one of the most abundant microRNAs in erythrocytes^30^ and its level is therefore perturbed even at low level of haemolysis^35,40^.

Haemolysis can be assessed by visual inspection of the samples or, more precisely, spectroscopically. An alternative approach is to compare the levels of the erythrocyte-enriched miR-451a and the haemolysis-insensitive miR-23a^20^. While visual inspection is rather subjective and not particularly sensitive, spectroscopic assessment and RT-qPCR quantification of miR-451a and miR-23a levels reveal even low degree of haemolysis^35^. Shah *et al*.^41^, compared several methods to assess the level of haemolysis in human serum samples and found ΔCq (miR-23a – miR-451a) to be the most sensitive indicator^41^. In contrast, Vliet *et al*.^13^ reported that absorption measurement is more sensitive for rat plasma samples. Our results show that the approaches are comparable and correlate well even at very low levels of haemolysis for all three sample types we tested (Fig. 4). An advantage of the qPCR approach is that haemolysis can be assessed even when the original sample is no longer available. The same strategy can be used to assess contamination with other cell-types when needed. For example, miR-425 level may reflect contamination of platelets^13^. It is important to be aware that the ΔCq (miR-23a–miR-451) indicator must be calibrated for every new biofluid, isolation procedure and RT-qPCR method, as the ratio of the measured levels of miR-23a and miR-451 depend on the relative bias introduced by the methods used^26,27,42^, but also the particular species and biofluids analysed. Indeed, in our study we concluded different threshold ΔCq values for the three sample types analysed. The ΔCq indicator should therefore be established for every workflow. Once calibrated the ΔCq indicator can be used to compare processed samples to identify outliers that should be reanalysed or discarded (see Supplementary file).

Despite several advances, circulating microRNA research has been hampered by inconsistency and poor reproducibility^1^. The Two-tailed quality control panel developed here is a simple yet powerful tool for researchers to optimize new workflows, assess the technical performance of an analysis, identify outlier samples, and generally improve the reliability of circulating microRNA data.

## Methods

### Oligonucleotides

Sequences of mature microRNAs were obtained from the miRBase release 22 (www.mirbase.org). RNA oligonucleotides with 5′-phosphate were synthesized and quantified by Integrated DNA Technologies. Spike-in miRNA sequences were screened *in silico* for homology against human, mouse and rat miRBase records (Release 22) with the following parameters - search sequences: mature miRNAs, search method: SSEARCH, e-value cut-off: 100, max. no. of hits: 100. No significant homology was found. DNA oligonucleotides were synthesized and quantified by Invitrogen. Sequences are available in Supplementary file.

### Samples

For the preparation of human serum, blood was collected from two healthy volunteers into 8.5 ml BD Vacutainer SST II Advance tubes (Beckman Dickinson) and allowed to clot for at least 30 min before centrifugation at 1500 g for 10 min at room temperature. The serum was then transferred to 2 ml tubes (Eppendorf) and stored at −80 °C. For the preparation of human plasma, blood was collected from four healthy volunteers into K_2_EDTA BD Vacutainer tubes (Beckman Dickinson) and centrifuged within 30 min at 1500 g for 15 min at room temperature. The plasma fraction was aspirated and transferred to 2 ml tubes (Eppendorf) and centrifuged again for 15 min at 3000 g. The supernatant was transferred to new 2 ml tubes and stored at −80 °C until analysis. Informed consent was obtained from all volunteers participating in the study. All procedures involving the use of human samples were performed in accordance with the ethical standards of Institute of Experimental Medicine, Academy of Sciences of the Czech Republic and with the Declaration of Helsinki. All methods were approved by the Ethical committee of the Institute of Experimental Medicine (decision on 22 June 2018, approval number 04/2018). For the preparation of rat serum, animals were anesthetized using 2–4% isoflurane. One millilitre of blood was collected from orbital plexus into 2 ml tubes (Eppendorf) using glass capillary. Blood was allowed to clot for 1 hour at room temperature and then centrifuged at 1000 g for 10 min. The clot was mechanically retracted from the tube wall before the centrifugation. Serum was transferred to another 2 ml tube and centrifuged a second time at 3000 g for 10 min. The supernatant was then transferred to cryovials (Biologix) and stored at −80 °C until analysis. All procedures involving the use of laboratory animals were performed in concordance with the European Community Council Directive of 24 November 1986 (86/609/EEC) and animal care guidelines approved by the Institute of Experimental Medicine, Academy of Sciences of the Czech Republic (Animal Care Committee decision on 17 April 2009; approval number 85/2009).

### Haemolysis dilution series

After whole-blood centrifugation, erythrocytes from the lower phase were collected into a separate tube and subjected to a freeze-thaw cycle followed by vigorous vortexing for at least 90 seconds to lyse the erythrocytes. The haemolysed test sample was prepared by adding 1% (v/v) of lysed erythrocytes into a non-haemolysed sample. A two-fold haemolysis dilution series was prepared by diluting the haemolysed sample sequentially with non-haemolysed sample. Dilution series from two subjects were prepared for each biofluid type (human serum, human plasma, and rat serum). Absorbance of free haemoglobin was measured at 414 nm, 540 nm, and 578 nm with a NanoDrop 2000 spectrophotometer (ThermoFisher) in duplicates. RNA was isolated from the serum and plasma samples as described below, starting with either 200 μl (human) or 150 μl (rat) input volume.

### RNA isolation

Total RNA was isolated from human plasma, and human and rat serum samples using the miRNeasy Serum/Plasma Advanced Kit (Qiagen) according to the manufacturer´s instructions. 1 μl of isolation spike-in mix containing synthetic cel-miR-54 (1e + 7 copies/μl), spike-A (2e + 5 copies/μl), spike-B (4e + 3 copies copies/μl) and, when appropriate 1 μl of GlycoBlue Coprecipitant (15 mg/mL) (Invitrogen), per sample was added at the lysis step. RNA was eluted into 20 μl nuclease-free water and stored at −80 °C.

### Reverse transcription and quantitative PCR

Reverse transcription (RT) reactions were performed with the qScript flex cDNA kit (Quantabio) in a total reaction volume of 10 μl. One reaction contained 2 μl of template RNA, 1x buffer, mix of 0.05 μM Two-tailed RT primers, 1 μl of GSP enhancer and 0.5 μl of RT enzyme, and nuclease-free water up to 10 μl. RT reactions were incubated in a CFX 1000 thermocycler (Bio-Rad) for 45 min at 25 °C, 5 min at 85 °C and then held at 4 °C. Immediately after incubation, cDNA was diluted by addition of 50 μl nuclease-free water. Quantitative PCR (qPCR) was performed in a total volume of 10 μl. One reaction contained 1x SYBR Grandmaster Mix (Tataa Biocenter), forward and reverse primer (final concentration 0.4 μM), and 2 μl of diluted cDNA template (resulting in a final cDNA dilution of 15x). qPCR was performed in duplicates and incubated in a 384-well plate in a CFX 384 Real Time Detection System (Bio-Rad) at 95 °C for 30 s, 45 cycles of 95 °C for 5 s, and 60 °C for 15 s followed by melting-curve analysis.

## Supplementary information

Supplementary file

## Data Availability

Cq values were pre-processed with CFX Manager 3.1 (Bio-Rad). Missing values were replaced with maximum Cq per assay + 1 (Cq_max_ + 1). Paired two-tailed T-test was used to calculate significance of difference of mean Cq values between extractions with and without glycogen and F-test was used to calculate significance of difference of spread of replicates (Fig. 3). For the calculation of F-test, Cq values were transformed to achieve normal distribution as: 2^ΔCq (Cq − Cq_mean_), where Cq_mean_ represents mean Cq of particular assay.
