# Supplementary material for: Two-tailed RT-qPCR panel for quality control of circulating microRNA studies
Source: Sci Rep. 2019 Mar 12;9:4255. doi: 10.1038/s41598-019-40513-w (PMC6414634; doi:10.1038/s41598-019-40513-w)
Supplement: Supplementary file 1 — Supplementary file [file 41598_2019_40513_MOESM1_ESM.pdf]

Two-tailed RT-qPCR panel for quality control of circulating microRNA studies

Peter Androvic, Nataliya Romanyuk, Lucia Urdzikova-Machova, Eva Rohlova, Mikael Kubista, Lukas Valihrach

1. Oligonucleotides and reagents

1.1 RNA and DNA oligonucleotides

Order following oligonucleotides from the oligo manufacturer of your choice:

<u>RNA oligonucleotides:</u>

Usage	Name	Sequence
Isolation spike-ins	cel-miR-54-3p	/5Phos/UACCCGUAAUCUUCAUAAUCCGAG
	miR-spike-A	/5Phos/UGCAGCCCUACCGACACGUUCC
	miR-spike-B	/5Phos/ACUCAGGUUGUAGGAGCGGUCUU
RT spike-ins	cel-miR-76-3p	/5Phos/UUCGUUGUUGAUGAAGCCUUGA
	cel-miR-2-3p	/5Phos/UAUCACAGCCAGCUUUGAUGUGC

Note: RNA oligonucleotides serve as miRNA spike-ins and should be ordered with 5´ phosphate modification to resemble endogenous miRNAs. 5´ phosphate modification also allows spike-ins to be ligated to the adaptors during preparation of small-RNA libraries for NGS, therefore they will be part of the final miRNA libraries. Ordering RNA oligos without 5´ phosphate modification will prevent this, although they still can be used to evaluate the isolation efficiency and inhibition of cDNA synthesis and PCR amplification via RT-qPCR.

<u>DNA oligonucleotides:</u>

Usage	Name	Sequence
Two-tailed RT primers	RT cel-miR-76-3p	AACAACGCACGCTCACAGACGTAGAGAACCTACGTCAACAATACATCAAGG
	RT cel-miR-2-3p	CTGGCTGAAACCACGCAGAGCTAGAGAACCTAGCTCAACACTACCGCACAT
	RT cel-miR-54-3p	AGATTACAAAGGACGCAGAGCTAGAGAACCTAGCTCAACACTACACTCGGA
	RT miR_spike_A	TAGGGCTAACAGAAACACAGCTAGAGAACCTAGCTGAACAATACAGGAACG
	RT miR_spike_B	ACCTGAGATGCGACACCCACCTAGAGAACCTAGGTGAACAATACAAAGACCG
	RT miR-23a-3p	TGGCAATGAATAGAACGAGAGCTAGAGAACCTAGCTCCTTAGAACCGGAAAT
	RT rno-miR-451-5p	AATGGTAAGCGGGTAAGACACCTAGAGAACCTAGGTGATAAATTGAAACTCA
	RT let-7a-5p	ACCTAACATGCTCTCCAGGTACAGTTGGTACCTGTCTCCACTTAACTAT
Forward qPCR primers	cel-miR-76-3p_FW	AACAACGCACGCTCACA
	cel-miR-2-3p_FW	CTGGCTGAAACCACGCAG
	cel-miR-54-3p_FW	GCAGATTACAAAGGACGCAGAG
	miR_spike_A_FW	GCTAGGGCTAACAGAAACACAG
	miR_spike_B_FW	CCTGAGATGCGACACCCAC
	miR-23a-3p_FW	GCTGGCAATGAATAGAACGAGAG
	rno-miR-451-5p_FW	CGAATGGTAAGCGGGTAAGACAC
	let-7a-5p_FW	ATGCTCTCCAGGTACAGTTG

Reverse qPCR primers	cel-miR-76-3p_RV	GGTTCGTTGTTGATGAAGCC
	cel-miR-2-3p_RV	GCTATCACAGCCAGCTTTGAT
	cel-miR-54-3p_RV	GCCTACCCGTAATCTTCATAATCC
	miR_spike_A_RV	TGCAGCCCTACCGACAC
	miR_spike_B_RV	CACTCAGGTTGTAGGAGCG
	miR-23a-3p_RV	GCATCACATTGCCAGGGATT
	rno-miR-451-5p_RV	CGCAAACCGTTACCATTACTGA
	let-7a-5p_RV	GCCGCTGAGGTAGTAGGTTGTA

Note: Desalting purification of DNA oligonucleotides is sufficient.

1.2 Reagents to be supplied

- qScript Flex cDNA Kit (Quantabio), cat. no. 95049-100
- TATAA SYBR® GrandMaster® Mix (Tataa Biocenter), cat. no. TA01-625, or similar

Note: Two-tailed RT-qPCR is optimized with qScript Flex cDNA Kit and TATAA SYBR GrandMaster Mix and all reference values reported here and in the main text of this manuscript are valid for the exact workflow described here. Other high-quality SYBR-green mastermixes are also expected to work.

2. Preparation of spike-in mixes

a. Dilute the lyophilized RNA oligo in the nuclease-free water or TE buffer. Calculate the number of copies of each miRNA spike-in per µl based on quantification readout from the oligo manufacturer.

   e.g. 100 nmole of lyophilizate contains 6.02E+16 microRNA copies. Dissolving lyophilizate in 60.2 µl of nuclease-free water will result in the solution with concentration 1e+15 copies/µl.

b. Prepare two working spike-in mixes according to tables below. One spike-in mix („isolation spike-in mix“) contains three RNA spike-ins in various concentration that serve as control for isolation efficiency. Second spike-in mix („Reverse transcription (RT) spike-in mix“) contains two RNA spike-ins that serve as control of the reverse transcription and PCR amplification. Mix together the individual RNA oligos to get the following final concentrations:

Isolation spike-in mix

RNA oligo	Final concentration (copies/µl)
cel-miR-54	1.00E+07
spike_A	2.00E+05
spike_B	4.00E+03

RT spike-in mix

RNA oligo	Final concentration (copies/µl)
cel-miR-76	1.00E+07
cel-miR-2	4.00E+03

Note: Avoid repeated freeze-thaw cycles of the spike-in mixes. Store them at -80°C in aliquots.

3. Experimental protocol

3.1 Usage of isolation spike-in mix to monitor the RNA isolation quality.

a. Mix 1 µl of isolation spike-in mix per 1 volume of the lysis buffer to be used for isolation of one sample. Prepare a mixture sufficient for all isolations including 10% surplus.

b. Use this mixture to lyse the samples. Immediately after addition of the lysis mixture to the biofluid sample, mix samples by vortexing to prevent exposure of spike-ins to the endogenous RNases.

c. Perform isolation procedure as recommended by the manufacturer.

3.2 Reverse transcription and usage of RT spike-in mix to monitor the cDNA synthesis

Multiplexed reverse transcription with Two-tailed RT-qPCR system is performed with a pool of target-specific primers with a unique secondary structure (for details see [1]). The Two-tailed quality control (QC) panel is comprised of eight Two-tailed primers targeting: i) three isolation spike-ins added before the RNA isolation procedure, ii) two RT spike-ins added before the cDNA synthesis, and iii) three endogenous miRNAs – haemolysis indicators miR-23a and miR-451a and let-7a miRNA, which is commonly present in serum and plasma samples.

3.2.1 Prepare a pool of Two-tailed RT primers:

a. Mix together following primers at the final concentration of 1 µM/each to create the RT-primer pool:

RT cel-miR-76-3p
RT cel-miR-2-3p
RT cel-miR-54-3p
RT miR_spike_A
RT miR_spike_B
RT miR-23a-3p
RT rno-miR-451-5p
RT let-7a-5p

Note: Avoid repeated freeze-thaw cycles of RT primer pool. Store it at -20 °C in aliquots.

3.2.2 Set up the reverse transcription reactions:

qScript Flex cDNA Kit (Quantabio) is used for cDNA synthesis.

a. Vortex and spin all components except the enzyme before use. Reverse transcription is run in a total volume of 10 µl per reaction. During the RT reaction set-up, use 1 µl of RT spike-in mix per RT reaction. If performing more than one RT reaction, prepare a mastermix including 10% surplus. Mix the following components in an RNase free tube:

	volume per one RT reaction (µl)	stock concentration	final concentration
Nuclease-free water	3.0*	-	-
RT primer pool	0.5	1 µM	0.05 µM
GSP enhancer	1.0	10 x	1 x
Buffer	2.0	5 x	1 x
RT spike-in mix	1.0	-	-
RT enzyme	0.5	-	-
RNA	2.0*	-	-

* Up to 5 µl of template RNA may be used per RT reaction with a total volume of 10 µl.

Note: We recommend running additional control reaction together with the samples:

"Spike-only" control *reaction contains all the RT reagents, however, instead of RNA sample, diluted isolation spike-in mix is used as a template. Isolation spike-in mix is diluted in nuclease-free water or the same buffer that was used for the final elution step of the RNA samples in the isolation protocol. Dilution factor is determined by the elution volume that was used during the RNA isolation procedure. E.g. if 1 µl of isolation spike-in mix was added before the isolation and RNA was eluted into 20 µl, dilute an aliquot of isolation spike-in mix 20x and use it as a template in "spike-only" control reaction. Add the same volume as the volume of RNA eluate used in RT reactions of experimental samples.*

The signal of the isolation spike-ins from the "spike only" sample represents theoretical 100% isolation efficiency and can be compared against signals of isolation spike-ins obtained from the samples. The approximate efficiency of extraction can be estimated from the formula:

$$Efficiency\ (\%) = \frac{1}{2\hat{}(Cq\ experimental\ sample - Cq\ \text{“}spike\ only\text{”}\ control)} *100$$

At the same time, the signal of the RT spike-ins from the "spike-only" sample serves as positive, contaminants-free control of the cDNA synthesis and can be compared against signal from the samples to evaluate the inhibition of the enzymatic reactions. Samples with a Cq difference >1 should be considered suspicious as they contain inhibitors of enzymatic reactions (see section 4. Interpretation of the data).

b. Briefly spin the contents and incubate:

Temperature	Time
25°C	45 min
85°C	5 min
4°C	hold

c. Dilute the cDNA by adding 50 µl of nuclease-free water directly to the RT reaction.

3.2.3 qPCR using SYBR mix

TATAA SYBR® GrandMaster® Mix (Tataa Biocenter) is used for qPCR.

a. Prepare qPCR assays for each target by mixing corresponding forward and reverse primer together at the final concentration 10 µM/each (for corresponding forward and reverse primers see section 1.1)

b. Prepare eight mastermixes - one for each miRNA to be analysed according to table below (calculate the amount needed for all samples including 10% surplus). We recommend running at least duplicate qPCR reactions per one cDNA sample.

	Volume (µl)	Stock concentration	Final concentration
Nuclease-free water	2.6	-	-
SYBR MasterMix	5.0	2 x	1 x
Primers	0.4	10 µM	0.4 µM

c. Dispense 8 µl of the mastermix into PCR-plate and add 2 µl of the diluted cDNA.

d. Seal and spin the PCR plate. Set the temperature and time profile in the real time PCR instrument. Set the reaction volume to 10 µl.

e. Incubate with following cycling conditions:

Initial denaturation*:	95°C	30 s	
40 cycles:	95°C	5 s	
	60°C	15 s	data acquisition
Melting curve:	65°C – 95°C		

* Refer to the manual of the particular SYBR mix used, initial denaturation time may differ for different mastermixes.

4. Interpretation of the data

4.1 Control for isolation quality and presence of inhibitors of enzymatic reactions

There are several possible sources of technical errors in a typical microRNA RT-qPCR workflow and Two-tailed QC panel is designed to reveal these errors on several levels and aid in troubleshooting. Several potential scenarios may occur, here we discuss their interpretation and suggested actions to take. We also refer reader to the decision tree in Figure 1 of the main text of this manuscript.

a. Compare the Cq values of the isolation spike-ins (cel-miR-54, spike-A and spike-B) between samples. Since these spike-ins were added in the same amount per isolation and the same volume of RNA eluate was used for cDNA synthesis of all samples, the Cq values should be comparable between samples. If so, their isolation efficiency was similar. The ΔCq values between individual isolation spike-ins should be between 3.5-5.5. Alternatively, if "spike-only" reaction was run together with the samples, its Cq value can be compared with Cq values of the samples. If the ΔCq of isolation spike-in assays (Cq experimental sample – Cq "spike only") is comparable between samples, their isolation efficiency was similar.

   If the Cq (or ΔCq) values of isolation spike-in assays of some samples deviate considerably from the rest of the samples (e.g. they have higher Cq by 2 or more), it is an indication of a technical problem during the workflow. Either the RNA isolation had lower efficiency than the rest of the samples, or the enzymatic reactions (reverse transcription and PCR amplification) were inhibited.

b. As a next step, examine the Cqs of RT spike-in controls (cel-miR-76 and cel-miR-2) to determine which step in the workflow was problematic (see below).

   - If the Cqs of isolation spike-ins and endogenous miRNAs show higher Cq in some samples, but the RT spike-ins have stable Cq across samples, it is an indication of the suboptimal RNA isolation. In such case, the affected samples should be re-isolated or excluded from the study.

   - If the Cqs (or ΔCqs) of the isolation spike-ins and also the RT spike-ins deviate significantly in the same samples from the rest (e.g. Cq of RT spike-ins is higher by 1 or more), it is an indication of presence of inhibitors of reverse transcription and/or PCR amplification. The effect of inhibition in standard samples can often be remedied by diluting the RNA eluate with nuclease-free water or TE buffer, so the contaminating substances are also diluted. However, this step also decreases concentration of the template RNA and therefore may not be optimal for samples with low RNA content, such as liquid biopsies. User can attempt to re-purify the RNA eluate, although this step will likely also lead to overall yield loss and may potentially bias the relative abundance of individual miRNAs. Samples affected by inhibition should therefore be re-isolated or excluded from the study.

c. If all of the samples have comparable Cq (or ΔCq) values of the isolation spike-ins, it suggests that all of the samples had similar isolation efficiency and there was no inhibition of reverse transcription and/or PCR amplification. As a next step, user should examine if the spike-B was detected in all samples. This miRNA spike-in is added at a very low concentration and therefore represents low-abundant miRNAs. Note that higher standard deviation observed with the low abundant targets such as spike-B is normal and even the complete absence of signal may be normal if high elution volume, low RNA input in cDNA synthesis or high dilution of cDNA was used.

- If spike-B is detected in all samples, the workflow worked well and user can proceed with the desired experimental measurement.
- If no deviation in two more abundant isolation spike-ins signals is observed (cel-miR-54 and spike-A) however spike-B miRNA spike-in is not detected in all or majority of the samples:
    - Examine the absolute value of ΔCq (Cq experimental sample - Cq "spike-only" control). The absolute value of ΔCq can be used to estimate the overall isolation efficiency from the formula:

$$\text{Isolation efficiency (\%)} = \frac{1}{2\text{\^{}}(\Delta \mathrm{Cq})} * 100$$

E.g. if the ΔCq is 1, approximately half the initial RNA content was lost during the isolation procedure (efficiency = 50%).

- If ΔCq is high (> 2-3 Cq) and spike-B is detected in "spike only" sample, it suggests that although isolation efficiency was similar between samples, overall absolute efficiency was poor and low-abundant miRNAs may have been lost. In such case, user should increase the RNA input and total volumes of reverse transcription and qPCR reactions, and is advised to consider using more efficient isolation protocol and check for RNase contamination.

- If ΔCq is low (< 2 Cq) and/or spike-B is not detected in "spike-only" control sample, it is an indication that low amount of isolation spike-in mix was added before isolation or that stock solution is degraded and contains lower amounts of spike-ins than expected. User can assume good quality of the samples as there are no signs of inhibition or low isolation efficiency, however the amount of isolation spike-in mix added should be increased in the next experiment and/or fresh stock solution should be prepared.

	Isolation spike mix			RT spike mix		Endogenous miRNAs						
	cel-miR-54	spike A	spike B	cel-miR-76	cel-miR-2	let-7a	miR-23	miR-451	Hemolysis indicator	ΔCq (spike-A - cel-miR-54)	ΔCq (spike-B - spike-A)	ΔCq (cel-miR-2 - cel-miR-76)
Sample 1	27.99	31.26	36.35	24.74	30.43	27.55	29.95	17.12	12.83	3.27	5.10	5.69
Sample 2	27.38	30.95	35.10	24.66	30.42	27.35	29.54	17.06	12.48	3.56	4.16	5.76
Sample 3	27.04	30.89	35.22	24.57	30.23	27.28	29.75	17.06	12.69	3.85	4.33	5.66
Sample 4	27.58	31.67	36.09	25.46	30.89	28.16	30.68	16.32	14.36	4.09	4.41	5.44
Sample 5	28.49	32.07	37.05	25.12	30.42	28.90	31.04	16.98	14.06	3.58	4.98	5.29
Sample 6	29.92	33.65	36.62	25.13	30.78	30.90	32.57	19.14	13.44	3.74	2.97	5.65
spike only ctrl	25.79	30.46	35.45	24.66	30.33					4.66	4.99	5.67

Example: *Here, one plasma sample was isolated in hexaplicate and quantified with Two-tailed QC panel. Often, problematic samples can be immediately identified by visual inspection of color-coded table of Cq values. Signal from isolation spike-ins in sample 6 deviates from others, while signal from RT spike-ins is comparable. This clearly suggest problems with RNA isolation. Apparent are also higher Cq values of endogenous miRNAs (note, that in this example, a technical replicate of the one biological sample is shown. There will be inherent biological variation in endogenous miRNAs between different biological replicates).*

	Isolation spike mix			RT spike mix	
	ΔCq (relative to spike-only control)				
	cel-miR-54	spike A	spike B	cel-miR-76	cel-miR-2
Sample 1	2.20	0.80	0.91	0.09	0.11
Sample 2	1.59	0.49	-0.35	0.00	0.09
Sample 3	1.24	0.44	-0.23	-0.08	-0.10
Sample 4	1.78	1.21	0.64	0.80	0.57
Sample 5	2.70	1.62	1.60	0.47	0.09
Sample 6	4.12	3.19	1.17	0.47	0.45

Alternatively, ΔCq values (relative to "spike-only" control) can be compared. Sample 6 deviates in ΔCq value of isolation spike-ins, while sample 5 can also be considered suspicious. All samples are free of inhibitors as demonstrated by low and comparable ΔCq values (relative to "spike-only" control) of RT spike-ins.

4.2 Control for haemolysis

Two-tailed QC panel contains assays for the miR-23a, miRNA with stable expression in plasma/serum samples not affected by haemolysis, and erythrocyte-enriched miR-451a. The Cq difference ($Cq_{miR\text{-}23a} – Cq_{miR\text{-}451a}$) reflects the level of haemolysis in the experimental sample [2] and can be used to assess haemolysis post-hoc, even when the original sample is not available anymore and standard methods such as spectroscopic measurement of absorbance of free haemoglobin at 414 nm cannot be used.

We have established reference values for human plasma, serum and rat serum. However, we advise users to establish their own standard curve based on standards similar to their experimental samples and their established workflow, as ΔCq values will be affected by other factors, such as sample origin, storage, processing and particularly isolation procedure. Therefore reference values provided here are valid for our workflow, but may differ with other experimental workflows and should serve as a rough guide, rather than an exact threshold.

a. Examine and compare the ΔCq values ($Cq_{miR\text{-}23a} - Cq_{miR\text{-}451a}$) between samples. Use reference values for non-haemolysed samples established here (based on Absorbance at 414 nm < 0.25) or from the own standards to infer the level of haemolysis in the original sample:

Sample	ΔCq ($Cq_{miR\text{-}23a} - Cq_{miR\text{-}451a}$)
Human plasma	< ~15
Human serum	< ~11
Rat serum	< ~6

If ΔCq of all samples is constant and below reference values, original samples had low and similar levels of haemolysis and are comparable. Samples with higher ΔCq had higher levels of haemolysis and should be marked as suspicious. Depending on the biological question they may still be used for the analysis (as long as the absolute levels of haemolysis are within reasonable boundaries – see above), as not all miRNAs are affected by haemolysis and studied effect may be higher than effect of haemolysis. However, any miRNAs of interest should later be validated for their sensitivity to haemolysis before any biological conclusions are made. Keep in mind that large variation in levels of haemolysis between samples may introduce a lot of noise into the data and removal of outlying samples should be considered.

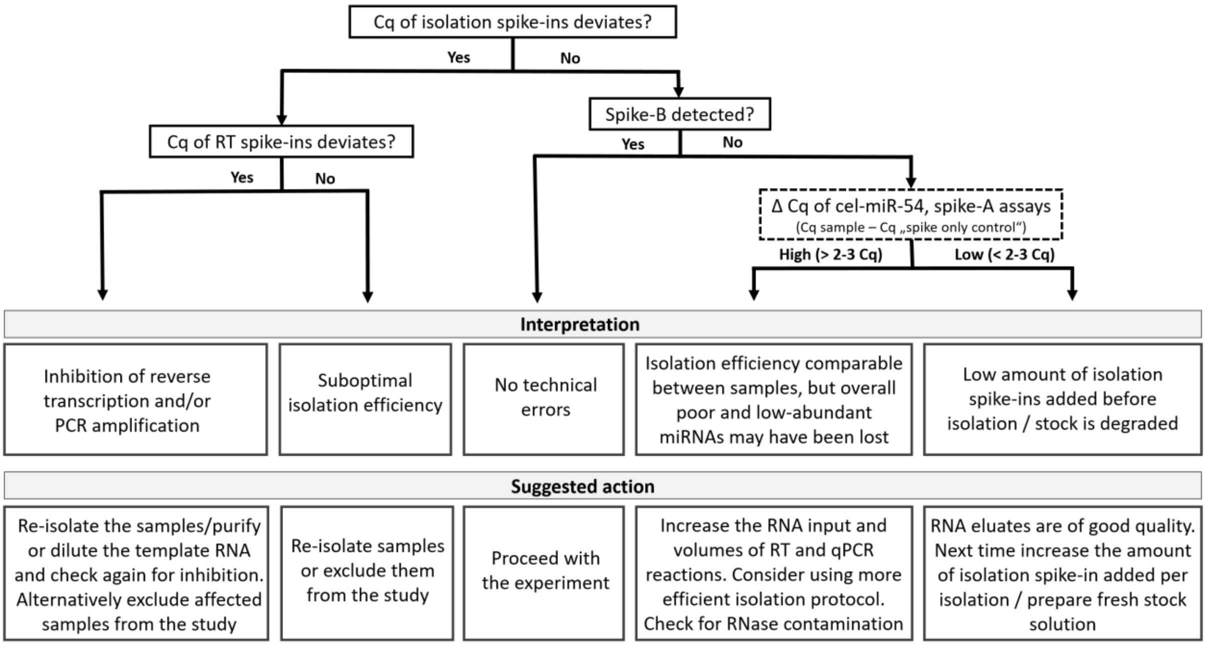

Figure 1B. Decision chart for the data interpretation and troubleshooting

References

1 Androvic, P., Valihrach, L., Elling, J., Sjoback, R. & Kubista, M. Two-tailed RT-qPCR: a novel method for highly accurate miRNA quantification. *Nucleic Acids Res* **45**, e144, doi:10.1093/nar/gkx588 (2017).

2 Blondal, T. *et al.* Assessing sample and miRNA profile quality in serum and plasma or other biofluids. *Methods* **59**, S1-6, doi:10.1016/j.ymeth.2012.09.015 (2013).
